# Preliminary Phytochemical Screening and Antispasmodic Activity of *Artemisia macrocephala* Jacquem

**DOI:** 10.4103/0975-1483.80300

**Published:** 2011

**Authors:** N Ali, SWA Shah, I Shah

**Affiliations:** 1 *Institute of Basic Medical Sciences, Khyber Medical University, Peshawar, Khyber Pakhtunkhwa; Department of Pharmacy, University of Malakand, Chakdara, Dir. Khyber Pakhtunkhwa, Pakistan*; 2 *Department of Pharmacy, University of Malakand, Chakdara, Dir. Khyber Pakhtunkhwa, Pakistan*

**Keywords:** Antispasmodic, *Artemisia macrocephala*, calcium channel blocking activity, folkloric use, verapamil

## Abstract

The current work describes the antispasmodic action of *Artemisia macrocephala*, which is achieved via blocking of the calcium channels. This explains its traditional use as an antispasmodic.The crude methanolic extract of *A. macrocephala* was studied for possible relaxant effect(s) on spontaneous rabbits’ jejunum preparations. Analytical-grade chemicals were used in the experimental protocols. *A. macrocephala* gave positive tests for flavonoids, saponins, glycosides, alkaloids, and terpenes. *A. macrocephala* caused relaxation of spontaneous rabbits’ jejunum preparations (*n*=6) at a dose of 10.0 mg/mL (EC_50_ = 6.95 ± 0.20 mg/mL; 95% CI: 6.2 to 7.5). Contractions induced by 80 mM potassium chloride (KCl) were also relaxed by the *A. macrocephala* at dose of 10.0 mg/mL. Attempting to find an explanation for the possible mode of action, we found that, *A. macrocephala* at concentration of 1.0 mg/mL produced rightward shift in the calcium chloride curves, with EC_50_ value of –1.65 ± 0.02 log [Ca^++^] M vs control with EC_50_ value of –2.44 ± 0.043 for calcium chloride curves. At a concentration of 1.0 mg/mL it could produce 52.4% of the control response at log [Ca^++^] M = –1.6. Similarly, verapamil at a concentration of 0.1 μM produced a rightward shift, with EC_50_ value of –1.74 ± 0.026 log [Ca^++^] M (95% CI: –1.66 to –1.82; *n*=6) vs control with EC_50_ value of –2.45 ± 0.05 log [Ca^++^] M (95% CI: – 2.23 to –2.91; *n*=6). The right shift of the EC_50_ values is justification for the folkloric use of *A. macrocephala* as an antispasmodic, suggesting that the possible mode of action is through calcium channel blockade.

## INTRODUCTION

*Artemisia macrocephala* (Synm: *Artemisia griffithiana* Boiss.) belongs to family Asteraceae.[1] The genus *Artemesia* has wide use as a phytomedicine. *Artemeisia judaica* is used in the treatment of itching, wounds, and congestion.[2] The leaves of *Artemisia stelleriana* Bess. are used as a carminative and in the treatment of peptic ulcer.[2] The hepatoprotective activity of *Artemisia maritima* has been also documented.[3] So far, essential oils that have been reported from *A. macrocephala* include α- and β-pinenes, camphene, Δ^3^ -carene, limonene, p-cymene,1,s8 cineole, camphor, and borneol.[4] Traditionally, fresh leaves of *A. macrocephala* have been used in the treatment of dysentery. The juice of fresh leaves of *A. macrocephala* are also used as an anti-allergic and antispasmodic.[1] Because of the many literature reports regarding the folkloric uses of *A. macrocephala* as an antispasmodic, we carried out our current work to scientifically study the species for its possible modes of action(s).

## MATERIALS AND METHODS

### Collection and identification of plant materials

The dried aerial parts of *A. macrocephala* was collected in the month of April–May, 2009, from the nearby hills (Badwan Chowk) of campus-I of the University of Malakand, Chakdara, Dir Lower, Khyber Pakhtunkhwa, Pakistan. The plant was identified by professor Dr Jehandar Shah, plant taxonomist and Vice Chancellor, Shaheed Benazir Bhutto University, Dir Upper, Sheringal. A voucher specimen, AM-01-2009, has been submitted to the herbarium of the University of Malakand.

### Preparation of methanolic extract

The shade-dried aerial parts of *A. macrocephala* were soaked in commercial-grade methanol and subjected to occasional shaking. After 15–20 days, the suspension was filtered. This process was repeated thrice. The filtrates were combined and concentrated under reduced pressure using a rotary evaporator at a temperature of 40°C. A greenish-black extract was obtained, which was used in the pharmacological screening(s).

### Drugs and animals

All the solutions were prepared in distilled water on the day of the experiments. Acetylcholine was purchased from BDH Chemicals, Poole, England. The rest of the chemicals were purchased from E. Merck, Germany. Rabbits of either sex were used in the experiments. The animals had free access to water. They were kept in fasting conditions 24 hours prior to the start of the experiments. The animals were treated as per the Animals Bylaws - 2008 of the University of Malakand (Scientific Procedures Issue-1), complying with international standards for handling the laboratory animals. The study protocol was duly approved by the legal bodies of the University of Malakand.

### Data recording

Graph tracings related to intestinal responses were recorded using an isotonic force transducer (Model No: MLT 0210/A Pan Lab S.I.) connected with PowerLab ^®^ (Model No: 4/25 T; AD Instruments, Australia) through a bridge pod amplifier. Other setting parameters were at range of 20 mV, low pass 10 Hz × 10 gain using input 1, rate 40/s.

### Interpretation of graph tracings, calculation, and statistical analysis

Chart 5 for Windows^®^ supplied with PowerLab^®^ was used to interpret the graph tracings. The mean EC_50_ values were calculated with the 95% confidence intervals. Student’s t test was used for assessing the significance of differences.

### Preliminary phytochemical screening

Preliminary phytochemical screening for the presence of terpenes, flavonoids, tannins, saponins, alkaloids, anthraquinone glycosides, and cardiac glycosides was performed as per reported procedures.[5,6]

### Effects on spontaneous rabbits’ jejunum preparations

Effects of *A. macrocephala* on spontaneous jejunum preparations were performed as per the procedure of Gilani *et al*. and a previous work of ours.[7,8] Rabbits were given a blow on the cervix to cause cervical dislocation. Their abdomens were opened. A portion of jejunum was isolated and maintained in a petri dish aerated with carbogen gas. Pieces of rabbit jejunum of about 1–1.5 cm in length were mounted in a tissue organ bath containing Tyrode’s solution. The tissues were allowed to become stable for at least 30 min. The crude methanolic extract was tested in concentrations of 0.01, 0.03, 0.1, 0.3, 1.0, 3.0, 5.0, and 10.0 mg/mL. The responses were recorded. The experiments were performed six times. Concentrations (mM) of the constituents of Tyrode’s solution were as follows: KCl 2.68, NaCl 136.9, MgCl_2_ 1.05, NaHCO_3_ 11.90, NaH_2_ PO_4_ 0.42, CaCl_2_ 1.8, and glucose 5.55. All the experimental protocols were carried out at 37 ± 1°C. Similarly, the effects on KCl (80 mM)-induced contractions in rabbits’ jejunum preparations were also recorded to clarify the possible mode of action. The rabbits’ jejunum tissues were maintained in a state of sustained contraction by KCl 80 mM in organ bath that was constantly bubbled with carbogen gas at 37 ± 1°C. The crude methanolic extract was tried in similar fashion as per reported work.[7,8]

### Effects on calcium channels

In order to explain the possible mode of action on rabbits’ jejunum preparations, we constructed calcium curves for the tissues treated with different concentrations of *A. macrocephala* in a decalcifying medium.[7,8] For confirmation of calcium channel blocking activity, the tissues were exposed to calcium-free Tyrode’s solution containing EDTA (0.1 mM). This was followed by experiments in potassium-rich Tyrode’s solution with the following composition (mM): KCl 50, NaCl 91.04, MgCl_2_ 1.05, NaHCO_3_ 11.90, NaH_2_ PO_4_ 0.42, glucose 5.55, and EDTA 0.1. Prior to this, all the tissues were stabilized in normal Tyrode’s solution for at least 30 min. Calcium response curves were constructed in the decalcified tissues with the cumulative addition of Ca^++^ at concentrations of 1 × 10^–4^ to 256 × 10^–4^ M.

## RESULTS

### Phytochemical screening

*A. macrocephala* tested positive for alkaloids, flavonoids, saponins, and terpenes. However, *A. macrocephala* tested negative for tannin, cardiac glycosides, and anthraquinone glycosides.

**Figure 1:**
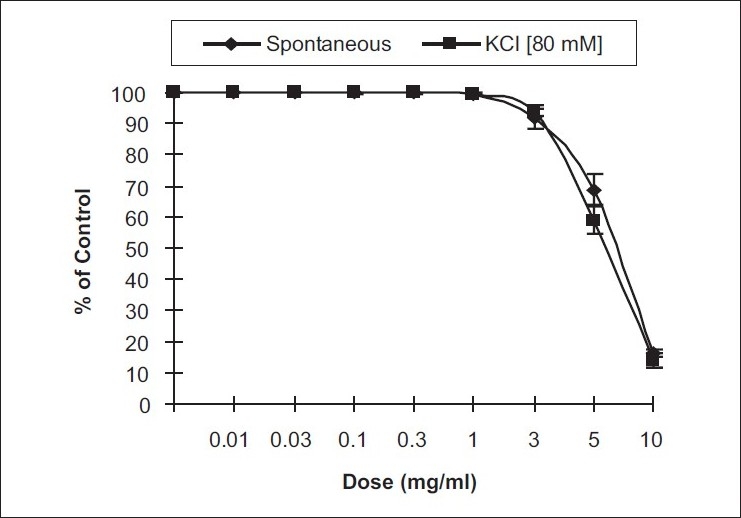
Relaxing effects of crude methanolic extract of *A. macrocephala* on spontaneous rabbits’ jejunum preparations and KCl-induced contractions

*Effects on spontaneous and KCl–induced contractions on rabbits’ jejunum preparations:* The effects of *A. macrocephala* are shown in Figure 1. *A. macrocephala* produced a relaxant effect on spontaneous rabbits’ jejunum preparations (*n*=6) with mean EC_50_ value of 6.95 ± 0.20 mg/mL (95% CI: 6.2 to 7.5). There was maximum relaxation at the concentration of 10.0 mg/mL. High-concentration (80 mM) KCl-induced contractions were also relaxed by *A. macrocephala* in a similar fashion. The effects on KCl-induced contractions and on spontaneous rabbits’ jejunum preparations are suprimposible [Figure 1].

### Calcium channel blocking activity

**Figure 2:**
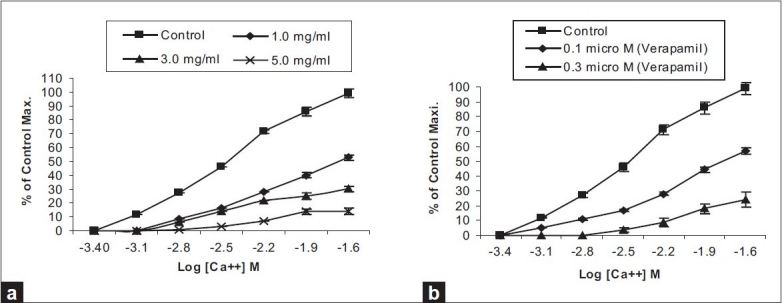
Calcium chloride curves in absence and presence of increasing doses of (a) Crude methanolic extract of *A. macrocephala* and (b) verapamil in isolated rabbits’ jejunum preparations

Positive relaxant effects by a test drug or an extract does not always indicate calcium channel blockade.[9] Plotting of calcium chloride curves helps confirm whether the mode of action is through the calcium channels. *A. macrocephala* at a concentration of 1.0 mg/mL produced a rightward shift in the calcium chloride curves [Figure 2a] with EC_50_ value of –1.65 ± 0.02 log [Ca^++^] M *vs* control, with EC_50_ value of –2.44 ± 0.043. *A. macrocephala* at a concentration of 1.0 mg/mL could produce 52.4% of the control response at log [ca^++^] M = –1.6. Similarly, verapamil at a concentration of 0.1 μM [Figure 2b] produced a rightward shift, with EC_50_ value of –1.74 ± 0.026 log [Ca^++^] M (95% CI: -1.66 to -1.82; *n*=6) vs control with EC_50_ value of –2.45 ± 0.05 log [Ca^++^] M (95% CI: - 2.23 to -2.91; *n*=6).

## DISCUSSION

In view of the traditional use of *A. macrocephala* as an antispasmodic, its extract was tested for possible inhibitory effects on spontaneous rabbits’ jejunum preparations. Positive relaxing effects on the spontaneous rabbits’ jejunum preparations confirmed its antispasmodic action. Positive relaxing effects on KCl-induced contractions suggested that the possible mode of action may be through calcium channel blockade.[7,8] There is usually an exchange of calcium between extracellular and intracellular stores. Moreover, the voltage-dependent calcium channels are responsible for the periodic depolarization and repolarization of the intestinal tissues that maintains its spontaneous contractions. Drugs that block these channels in the intestines will have a relaxing effect.[10–13] Plotting of calcium chloride curves in the extract-treated tissues proved that the relaxing effects are through calcium channel blockade. Comparison with the curves of calcium chloride in verapamil-treated tissues confirmed that the mode of action of the extract was through other calcium channels because of the right shift in the EC_50_ values.[14]

## CONCLUSION

Thus, we conclude that *A. macrocephala* has an antispasmodic action through the calcium channel blocking mechanism, which justifies its folkloric use as an antispasmodic. The antispasmodic action may be attributed to the presence of active phytochemical constituents in the plant. Further work is necessary for activity-guided isolation of the pharmacologically active substances.
